# Monitoring Colonies of Large Gulls Using UAVs: From Individuals to Breeding Pairs

**DOI:** 10.3390/mi13111844

**Published:** 2022-10-28

**Authors:** Alejandro Corregidor-Castro, Marie Riddervold, Thomas Eske Holm, Thomas Bregnballe

**Affiliations:** 1 Department of Ecoscience, Aarhus University, C.F. Møllers Allé 8, DK-8000 Aarhus C, Denmark; 2 Dipartimento di Biologia, Università di Padova, Via U. Bassi 58/B, I-35131 Padova, Italy

**Keywords:** conservation, drone, UAVs, breeding birds, monitoring

## Abstract

Measuring success or failure in the conservation of seabirds depends on reliable long-term monitoring. Traditionally, this monitoring has been based on line transects and total or point counts, all of which are sensitive to subjective interpretation. Such methods have proven to consistently record fewer individuals than intensive efforts, while requiring many hours of fieldwork and resulting in high disturbance. New technologies, such as drones, are potentially useful monitoring tools, as they can cover large areas in a short time, while providing high-resolution data about bird numbers and status. This study conducted two types of Uncrewed Aerial Vehicle (UAV) surveys in a big colony of multispecies breeding gulls. From a 25 m height, we photographed 30 circle plots where nests were also counted on the ground, showing that the number of occupied nests/breeding pairs could be estimated accurately by multiplying the number of counted individuals with a 0.7 conversion factor. A fixed-wing UAV was used to photograph the entire island to compare drone counts with counts conducted by traditional methods, were we counted a higher number of breeding pairs than the traditional count (1.7–2.2 times more individuals). It was concluded that UAVs provided improved estimates of colony size with much reduced monitoring effort.

## 1. Introduction

Monitoring long-term abundance of breeding seabirds provides the foundation for effective conservation-based research [1], as well as appropriate habitat management by observing population trends [2,3,4], providing the necessary insight to identify and tackle conservation problems at an early stage [5]. The evaluation of success or failure of conservation strategies thus depends on precise documentation of how populations of the target species develop in response to the actions taken [1].

Conventional monitoring of breeding seabirds uses a variety of methods, such as line transects [6,7,8] and ground counts [9,10,11]. These require many hours of fieldwork and familiarity with the area [8], as well as a constant human presence during the course of the counts. This may cause high levels of disturbance to breeding birds, affecting individual breeding success, or even causing colony failure [12,13,14]. In addition, use of these traditional methods comes with multiple sources of associated error and variance [15], which may be difficult to quantify. For instance, decreases in observer detection rates due to hearing loss with advancing age [16], systematic increases (up to 4.3%) in counts between initial and subsequent surveys (the so-called “first-time observer effect” [17]). Correction for declining avian detection probabilities with sampling distance [6,18] or when monitoring cryptic species [19] may also need to be considered. Experience shows that these factors result in underestimation of true abundance: traditional census methods consistently recorded fewer breeding pairs than other more intensive studies, rounding a 30% less detection of the total numbers [6,20], and up to a 50% reduced detection [11,21,22]. Having resolved these sources of bias and error, there remains the challenge of converting the numbers of birds detected at the colony to the true numbers of breeding pairs. This is usually achieved by applying a correction factor to the counts, but this needs to reflect local breeding conditions, such as habitat composition and density [1,7] and is typically specific to each species [11]. For example, for counting breeding pairs of seabirds in the Wadden Sea, Hälterlein et al. [23] proposed a standard factor of 0.7 to correct for the effect of arbitrary use of the methods.

New technologies such as Uncrewed Aerial Vehicles (UAVs, popularly known as drones) are potentially useful alternative monitoring tools, and their use has increased in recent years [3,12,24,25,26,27,28]. Drones can survey large areas in short duration flights [29,30] in inaccessible or dangerous areas [31,32], providing high-resolution data and accurate information about numbers of birds and various breeding parameters [13,33,34], while minimizing or even eliminating disturbance to birds (e.g., [3,29]). Traditionally, the use of these technologies required a high processing time due to the need for humans to manually derive counts from imagery [2,32], which in some cases negated efficiency gains from data collection [3,35]. Nonetheless, proposed flying methodologies and new approaches to image analysis have been developed [26,28,36] reducing these processing times up to eight-fold [35]. Therefore, UAV’s are a potentially good alternative to traditional counts in terms of efficiency, time investment and financial cost.

The aim of this study was to determine the advantages and disadvantages of the use of UAVs as a monitoring tool to census colonies of two species of breeding seabirds, the Herring Gull (*Larus argentatus*) and the Lesser Black-backed Gull (*Larus fuscus*); specifically regarding our prediction that traditional methods may carry a bias due to their subestimation of breeding pairs. The studied colonies, holding almost 20,000 individuals, are located on the island of Langli in the Wadden Sea. To determine the relationship between the number of nests and individuals observable on aerial photos, we compared the outcomes from the UAV surveys with detailed counts of nests on the ground inside small-scale plots. Since the number of individuals inside the colony area might vary throughout the day, the timing of photographing could potentially be important, and therefore, we studied the temporal variation in the number of individuals present in a part of the colony. We also compared the output from aerial counts on a large scale (full island survey) with results produced from traditional counting methods, where the number of individuals inside the colony area were counted from vantage points on the ground. With this, and based on our identification of an appropriate conversion factor, we propose a standardized way to efficiently monitor large gull colonies.

## 2. Materials and Methods

### 2.1. Study Site

We conducted the study in May 2018 on the 80 ha. island of Langli (55°30′ N, 8°18′ E), in Ho Bay, the northernmost island in the Wadden Sea. Approximately 2 km long and 0.5 km wide at its widest; this island is uninhabited and only open to the public outside of the breeding season between mid-July and mid-September. There was limited human activity on Langli during 1983–1999 associated with a year-round manned field station, but since its closure, humans have only disturbed breeding birds during 3–4 visits to annually monitor them. The island consists of dunes in its central region and marsh areas on the northern and southern ends, with abundant common velvet grass (*Holcus lanatus*) and red fescue (*Festuca rubra*). Wetter areas are dominated by common saltmarsh grass (*Puccinellia maritima*) and sea lavender (*Limonium vulgare*). The island is part of an internationally important protected area for waterbirds under the Ramsar Convention and the EU Birds Directive. Herring Gull (HG) numbers increased from 250 pairs in 1971 to 1100 pairs in 1983, and increased again after 1993 to reach around 9900 pairs in 2015, after which numbers have fluctuated ([37] and unpublished data). The Lesser Black-backed Gull (LBBG) began breeding on Langli in 1990 and numbers increased to c. 300 pairs in 1999, and to 2000 pairs in 2011, but numbers declined to 1100 pairs in 2020. Both species of large gulls often breed in mixed colonies [38], as is the case on Langli. The Danish populations of HG and LBBG are estimated at c. 90,000 and 5000 pairs, respectively [37]. In 2012, the total number of HG in the international Wadden Sea reached 60,000 pairs and the total number of LBBG was 94,000 pairs [39].

### 2.2. Field Procedures

**Figure 1:**
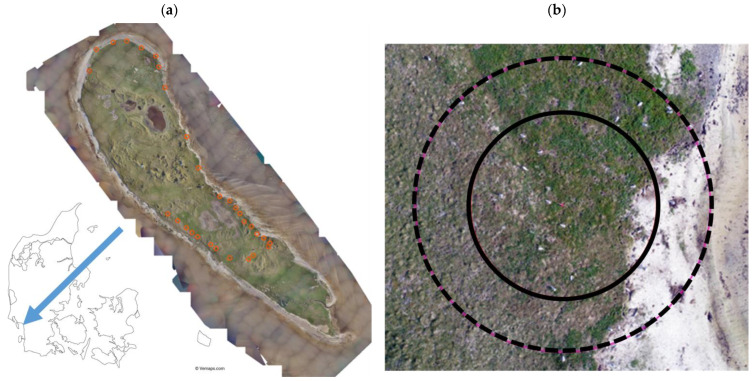
(**a**) Location of the 30 circle plots (in orange) on the island Langli. Image taken with the E384 fixed-wing UAV (TEH). (**b**) Circle plot number 21. The pink line shows the 11 m radius, whereas the red line shows the 7 m radius. Image taken with a DJI Phantom 4 Pro.

To conduct nest counts, we placed 30 circle plots scattered around the island (Figure 1a). We placed these plots along lines in order to locate them more easily when flying the copter drone. We placed a red or yellow cone (with a number between 1–30) in the center of each plot for increased detectability, and different colorful markers at 7 and 11 m radius (two markings on the southern perimeters and two markings on the northern perimeters) to geo-reference the aerial photos (Figure 1b). We obtained the GPS location of each circle plot using a Normad 1050 (Trimble ^®^, Sunnyvale, CA, USA).

We conducted surveys two weeks apart to record the number of individuals present at the incubation stage (14–15 May), and once chicks started to hatch (29–30 May). On 14 and 29 May, three observers counted all the nests within the 30 circle plots, using a rope marked at 2, 7, and 11 m distances, attached to the plot center. When the observers walked the circle, one observer was located at the 2 m mark (counting all the nests from 0–2 m), one at the 7 m mark (counting from 2–7 m), and one at the 11 m mark (counting from 7–11 m). We recorded both the number of nests and the number of eggs they contained (0, 1, 2, 3, and >3 eggs). In addition, we noted if the nests contained chicks, and how many. Unfortunately, it was not possible to distinguish HG nests from LBBG nests with certainty during the nest counts, so we corrected this by using the ratio of observed HG/LBBG in the breeding areas per circle plot, and applying this ratio to the observed number of nests. On the mornings of 15 and 30 May, we conducted two other aerial surveys (two each day; four in total). On each day, the first survey consisted of flying a DJI Phantom 4 Pro (66 db), with a standard camera lens FOV 84° 8.8 mm/24 mm f/2.8-f/11, at 25 m over the circle plots. Including a change of the batteries, the total flight time did not exceed one hour.

The second survey consisted of a flight over the entire island using an E384 fixed-wing UAV with a Sony ILCE-QX1 camera. On 15 May, this full island survey was conducted at a 75 m height. The wind speed was around 2 m/s (from the south-east) and the UAV flew at 13 m/s. The flight started at 08:00 am (GMT + 2) and took approximately two hours. The flight was repeated on 30 May, but at 40 m altitude. The wind speed was 7 m/s (from the west), and the speed of the UAV was 13 m/s. Again, the flight started at 08:00 am (GMT + 2) and was slightly over 2 h duration. In none of the flights was it necessary to stop for a battery change. On 28 May and 2 June, experienced surveyors conducted the traditional ground counts of individuals using the sand dunes as vantage points. This method has been used since 1998 (19 years), and we used the data from these previous surveys to assess the range of variation in ground counts. All flights started at least 150 m away from the closest nest, in order to avoid disturbance at take-off.

### 2.3. Nest Survey

**Figure 2:**
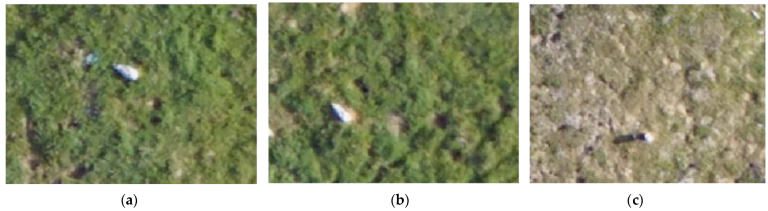
Images representing the different behaviors perceived during the manual identification of the individuals: (**a**) HG categorized as lying down (sitting), without a visible nest nearby; (**b**) HG lying down on a nest (incubating), based on the presence of nesting material under the individual; (**c**) LBBG standing, based on the presence of a long shadow coming from the individual.

The images from the DJI Phantom 4 Pro were loaded and geo-referenced into ArcMap 10.5 [40]. We drew the 11 m circles on the images using the visible marks to define the periphery of each. Five images from the first flight (15 May) did not achieve full coverage of the 11 m circle. Gulls and nests located on the exact rim of the 11 m circle were disregarded. In order to avoid double counting and improve counting efficiency, we used the point function in ArcMap to mark all the visible birds and nests within the plots. We identified all the birds to species level, categorizing them as “unknown” in case of doubt. We identified groups of nesting Common Gulls (*L. canus*, CG) by their smaller size compared to HG by using the measurement tool in ArcMap to determine the size of each individual. Thus, a gull was categorized as a HG if it was larger than 0.4 m, and as a CG if it was shorter than 0.4 m [41]. We allocated each nest/individual in the nest to one of the following four behavioral/nest categories: empty/unattended nest (nests); bird lying down (sitting) (Figure 2a); bird lying down on a nest (incubating), based on the presence of nesting material under the individual (Figure 2b); bird standing, based on the presence of a long shadow coming from the individual (Figure 2c); behavior unknown (un).

As a first step, and in order to test the validity of applying the conversion factor of 0.7 suggested by Hälterlein et al. [23], we used linear regressions (significance level of 0.05) to test for the relation between nest numbers recorded on the ground (dependent variable) and the numbers of individuals recorded on the photos (independent variable). The relation was compared with different conversion factors ranging from 1 (original count) to 0.4. Then, we used a similar approach to assess the most efficient method to estimate the number of breeding pairs in the area using: (i) the sum of birds categorized as lying down on a visible nest (incubating) + unattended nests (nests), (ii) the sum of birds categorized as lying down on a visible nest (incubating) + unattended nests (nests) + birds categorized as lying down (sitting), i.e., all the birds seemly behaviorally nesting, (iii) the sum of all behavioral and nest categories (incubating + sitting + nests + un), (iv) the sum of all behavioral and nest categories (incubating + sitting + nests + un) multiplied by the 0.7 conversion factor suggested by Hälterlein et al. [23]. As a dependent variable, we chose the number of nests observed during the ground count corrected per species. We considered as best models the ones with better values of Adj R^2^ and slopes closest to 1. We performed the statistical analysis with R-studio software 3.5 [42,43].

### 2.4. Diurnal Variation

To minimize the noise inherent in comparing count results from different years and localities, it is relevant to know whether the relationship between the number of nests and the number of individuals present within a colony area varies during the day. This might be the case when partners not engaged in incubation fly out to forage at a certain time of the day and more or less simultaneously return. To study the extent of diurnal variation in the number of individuals present inside the colony, we installed wildlife cameras on 5 m high poles next to a plot with nesting HG and next to another plot with nesting LBBG. Pictures were taken at 20 min intervals between 04:20 and 12:00 h, between 30 May and 8 June. To limit the survey to days before eggs started to hatch and to days without disturbance, we only included pictures taken on 31 May and 1 June at the HG plot and only data from 31 May and 1 and 2 June at the LBBG plot. On each picture, we counted the number of individuals present within the defined area covered by each plot. We tested whether the number of birds present varied from early morning to midday. We applied a model where we corrected for a first-order autocorrelation (i.e., some of the individuals photographed were identical to those photographed 20 min. earlier) and we included date as a random effect. The models were calculated using proc mixed in SAS version 9.4 [44]. To visualize the temporal change in the number of individuals present, as well as in the variance (from picture to picture) in numbers present, we divided the morning into 2 h periods.

### 2.5. Full Island Survey

The images from the two full island surveys conducted by the E384 fixed-wing (15 and 30 May) were stitched with the program Drone2Map from ESRI [40]. Based on the stitched photos for the full island survey, all birds and visible nests were counted manually in ArcMap, using a similar criteria as for the circle plots. The survey photos taken on 30 May did not result in a complete coverage of the entire island, as the camera ran out of battery. Because of this, the numbers present in areas that were not covered (3 out of 79 ha, 3.8%) were extrapolated from the total counts of the rest of the island (i.e., the proportion and number of each species for that specific area was calculated, comparing the rest of the island for both dates). In total, it took approximately 75 h to manually count and behaviorally categorize all the birds present on the island for each full survey. On both surveys, there were areas with very poor-quality resolution, making some of the birds look blurry (as part of the stitching process). In these cases, the birds were not assigned behavioral states. We excluded birds located in the water, but included individuals on the sand within 25 m of the official coastline.

## 3. Results

To evaluate the validity of the method suggested by Hälterlein et al. [23] (breeding pairs = numbers of individuals present in the colony multiplied by 0.7), we used linear regressions (significance level of 0.05) to identify the relationship between the number of individuals present on photos taken from the UAV and numbers of nests recorded on the ground. The statistical parameters for the different linear models fitted supported the suggestion to use the value 0.7 when converting from numbers of individuals to numbers of occupied nests (Table 1). This was the case for the HG in the first flight (F = 69.93, df = 1 and 23, *p* < 0.001) and for the LBBG in both flights (F = 608.3, df = 1 and 23, *p* < 0.001; F = 4989, df = 1 and 28, *p* < 0.001). For the HG in the second flight, a conversion factor of 0.6 was slightly better supported than the conversion factor of 0.7 (F = 216, df = 1 and 28, *p* < 0.001).

**Figure 3:**
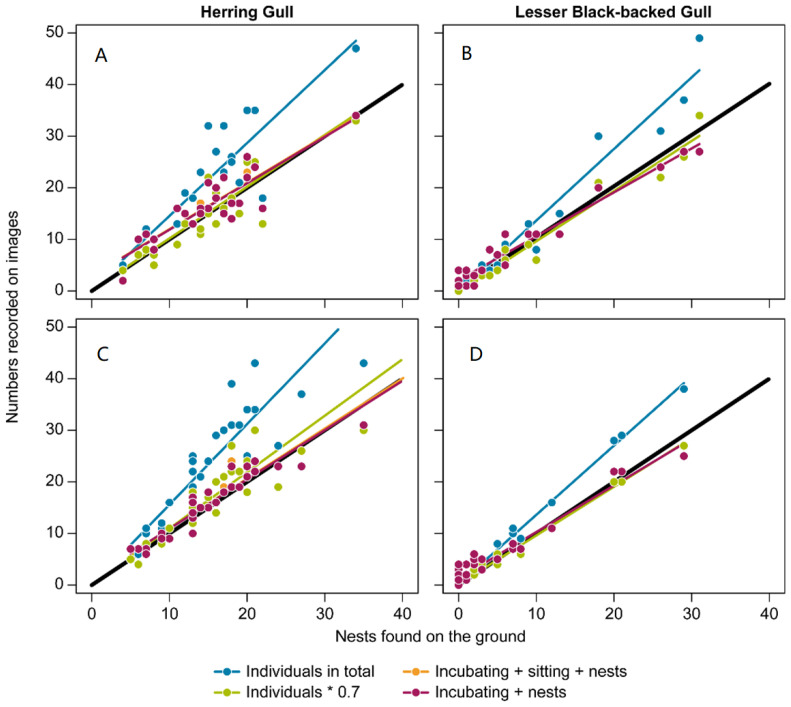
Linear models showing the relation between the number of nests found inside the circle plots and the number of gulls counted on photos taken from the drone on 14 and 15 May (**A**,**B**) and 29 and 30 May (**C**,**D**). Values for the Herring Gull (HG) are represented in graphs (**A**,**C**), whereas values for the Lesser Black-backed Gull (LBBG) are represented in graphs (**B**,**D**).

The analysis of the different linear regressions for the circle plots is shown in Figure 3. For the HG in the first flight (15 May), the model using the conversion factor of 0.7 presented the best fit in terms of Adj R^2^ (0.74) and slope (0.99; F = 69.93, df = 1 and 23, *p* < 0.001). This relationship changed when the second flight was conducted two weeks later on 30 May. On that date, the model considering only the behaviors nests + incubating + sitting fitted the counts better (F = 453.6, df = 1 and 28, *p* < 0.001), with an Adj R^2^ of 0.94 and a slope of 0.97. For the LBBG, there was no difference between dates with respect to the conversion factor of 0.7 required to obtain the best fit (F = 608.3, df = 1 and 23, *p* < 0.001; F = 4989, df = 1 and 28, *p* < 0.001), with an Adj R^2^ of 0.96 and 0.99, and a slope of 0.97 and 0.96, respectively.

**Figure 4:**
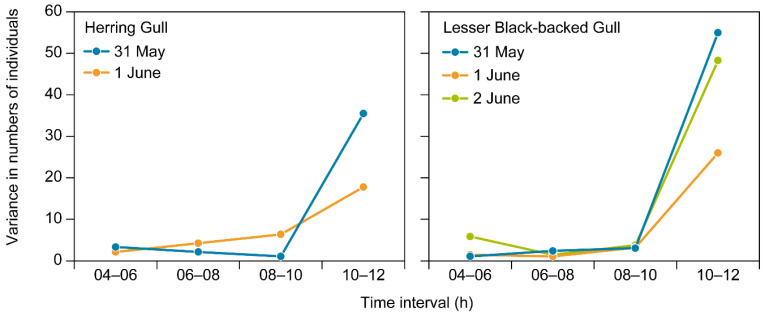
Variation in numbers of Herring Gulls (**left figure**) and Lesser Black-backed Gulls (**right figure**) present inside the two study plots from early morning to midday given for the four 2-h intervals on 31 May and 1 June (HG) and 31 May–2 June (LBBG).

The mean number of individuals present inside the two plots photographed with wildlife cameras slightly declined, but not significantly, from early morning to midday (Table 2 and Table 3; the decline amounted to 8–9% from the first two hours of the morning to the last two hours before noon). More markedly, the number of individuals present inside the plots began to extensively vary during the last hours before noon, whereas numbers were far more stable during the hours between 4 am and 10 am (Figure 4, Table 2). This suggests that UAV surveys of colonies of the two study species should be carried out before 10 am.

The number of birds observed during the two fixed-wing flights (15 and 30 May) and the number of individuals counted in both ground counts is given in Table 4, together with the estimated number of breeding birds based on multiplying the number of individuals by 0.7. It is evident that the estimated number of breeding pairs, based on the photos taken, was 1.7 times higher for the HG and 2.2 times higher for the LBBG. In addition, and similar to earlier years, we observed within year differences in numbers recorded during the traditional counts in 2018. The average discordance between the maximum numbers recorded (here set as 100%) in the years 1998–2019 and numbers recorded on other earlier or later dates within the season was 19.7 ± 12.2% (SD) (min. 3.3 and max. 44.9%) for the HG, and 26.9 ± 18.7% (min. 1.6 and 60.1%) for the LBBG.

## 4. Discussion

In this study, we determined that, among all the possible correction factors, the applied standard factor of 0.7 proposed by Hälterlein et al. [23] gives the most precise conversion from numbers of counted individuals present in the colony area to an estimate of the number of breeding pairs with nests. For the first flight (15 May), the model using a conversion factor of 0.7 proved to be the best fit for both the HG and the LBBG. While this trend continued for the LBBG in the second flight (30 May), the best fitted model for the HG for this period was the one using a conversion factor of 0.6. However, it is important to note that this fit was just slightly better than a conversion factor of 0.7, and thus should be interpreted with caution. An explanation for this could be that, on 30 May, some HG eggs had started to hatch, and thus the HG adults were more present at the nest. This possible explanation is supported by the models fitted for the two different periods. While, for the first period (Figure 3), the best fitted models were the ones considering a conversion factor of 0.7 for both species, the best fitted model for the HG in the second period was the one considering only the behaviors related to nest proximity (incubating, nests, and sitting). This suggests a higher recording of individuals in the breeding grounds behaviorally not nesting, thus leading to an overestimation when using the 0.7 factor. In addition, as LBBG eggs hatch later, the 0.7 conversion factor was still the most efficient on these dates. Much time was expended to record the individual behavior of each individual detected in the photo. For this reason, we strongly recommend conducting the surveys before the eggs start to hatch, and only count numbers of individuals observed in the imagery, instead of accounting for the behavior of each individual, prior to applying the conversion factor of 0.7 proposed by Hälterlein et al. [23].

In this study, we also determined that UAVs were effective tools to monitor and precisely estimate the size of large gull colonies. First, the use of the drone reduced the time required to conduct the survey [45]. This is of great importance, as surveys are often conducted in challenging terrain, using expensive manpower at considerable cost and disturbance [31,32,45]. Moreover, UAVs can also provide a significant increase in detection probability. According to Chabot et al. [25], it took 12 surveyors around 4 h to count all nests in a large Common Tern (*Sterna hirundo*) colony (48 “working hours”), whereas the total time for two operators to fly the UAV, including set-up and pack-up, was no more than 90 min (3 “working hours”). Based on the estimations of [7] and [8], an intensive survey of our study area (80 ha) would have taken approximately 375 h. This exceeded the sum of the total flight time (around 2 h) and our unnecessarily detailed processing of the images (75 h, including the time required to identify the posture/behavior of each individual). In addition, and of great importance, we detected far more breeding birds when using the UAV to cover the entire island. The recorded number of individuals by the traditional method provided close to half the numbers recorded by the use of the UAV. These numbers are similar to the differences observed between traditional counts and more intensive surveys (see [11,20,22,23,31]). Overall, our results support the use of drones as a more efficient, more precise, and less time-consuming tool for monitoring ground-nesting colonial waterbirds and seabirds. In addition, our analysis of the ground surveys conducted for over 19 years showed that the counts carried out 2–3 times per season varied by around 20% for the HG and 27% for the LBBG. This variance was higher than that obtained during the UAV surveys (5% for the HG and 15% for the LBBG), suggesting that UAV surveys help reduce the amount of uncertainty when estimating total numbers of breeding pairs. Nonetheless, it is worth mentioning that we detected a lower number of individuals in the second flight, which may reflect a lower amount of breeding individuals in the area (i.e., breeding failure). We do not think the drone flight itself may have accentuated this failure, but systematic studies investigating this ought to be conducted.

UAVs also helped to solve one of the general concerns with traditional monitoring methods, which is the disturbance caused towards the birds. Gulls are generally prone to flush when humans are present, leaving the nest unattended and vulnerable to predation for long periods of time, but they do not exhibit the same reaction to UAVs [31]. Several studies have tested for behavioral responses to UAV surveys. In their study, Rush et al. [26] flew a small UAV as low as 15 m above a Lesser Black-backed Gull colony, and described the behavioral impact on the birds as low to negligible. We have had the same experience when applying drones in our monitoring of four species of gulls at nine different localities in Denmark [46,47]. Moreover, Vas et al. [48] investigated the disturbance reaction to UAVs with three species (Mallard *Anas platyrhynchos*, Greenshank *Tringa nebularia*, and Great Flamingo *Phoenicopterus roseus*), and they were able to fly as close as 4 m without the birds showing any sign of disturbance. Thus, UAVs can be used for surveying a range of bird species without disturbance being an issue. However, the presence of raptors in the survey area does seem to affect bird reactions to the UAV, and it is therefore advisable to test for these possible agonistic/distress reactions before doing full UAV flights [31]. In this study, while there was no systematic measurement of disturbance, we noticed that the DJI Phantom 4 Pro did not incite the nesting birds to flush. The only time we observed a response behavior was when the UAV made a quick up-flight, causing its propeller to increase speed and noise. The E384 fixed-wing drone caused the birds near to flush only during take-off. Once the UAV was in the air, no further disturbances were observed. In general, the view of UAVs is that they significantly reduce or cause no disturbance of birds [45].

Nonetheless, the use of UAVs does present some downsides. First of all, many models of UAVs are sensitive to weather conditions [25]. This means that long periods of bad weather, such as rain or high wind episodes, may inhibit the use of drones to perform population surveys, requiring flexibility and planning from the surveyor. Another issue may be related to data collection. In this study, we experienced some problems when using the copter drone to record specific areas or transects, as we often found it difficult to see if the drone captured all the planned locations on the live video. Incomplete coverage of key selected areas may not have been evident at the time of survey, resulting in loss comparisons with ground counts of individuals. For example, some of our circle plots where not fully covered during the survey on 15 May due to the elevation changes across the island. A third issue was related to the stitching process of the images. Transect flights are based on the continuous capture of images that overlap with each other, and are stitched together in order to provide an orthomosaic of the full transect. Because this stitching process cannot be conducted in the field, errors related to single images or specific parts of the transect may not be discovered until the surveyor has left the area. One solution to this problem would be to repeatedly sample the same location, which would also provide a more precise estimate [49,50]. Lastly, one of the most prominent disadvantages with UAVs is the time-consuming data analysis process. Currently, there is an increased interest in supporting the use of automated counting methods using image recognition software, which if reliable, would mean a noticeable reduction in data analysis time [12,26,51]. Thus, it is important to work on methodologies that would allow for automatic ways of counting and processing the data, so the efficiency of UAVs outweighs traditional methods of surveying. In the present case, the time required to process the orthomosaics could have been reduced by approximately 20% of the time actually used if we had focused only on counting individuals and not attempted to record the nests and posture/behavior of each individual. We learned that this, in combination with applying the conversion factor of 0.7, would have been a sufficient method to estimate the size of the colonies of the two species.

## 5. Conclusions

This study supports the use of the conversion factor from numbers of individuals to numbers of breeding pairs proposed by Hälterlein et al. [23]. This procedure gave an even more precise estimate than trying to identify the presence of nests and postures of each of the individuals identified on the photos. We also found that it was possible to use different UAVs to get a precise estimation of the size of large colonies of Herring Gulls and Lesser Black-backed Gulls. Despite birds needing to be excluded from the estimates (due to poor photo quality after stitching, or high vegetation cover), the aerial count was still able to provide substantially higher numbers of breeding birds than the ground surveys, and the uncertainty of count results was lowered. This led us to the conclusion that UAVs can provide researchers with more valid information about the sizes of colonies. We also found that it is recommendable to conduct UAV surveys of the two study species before 10 am, to minimize temporal variation in the number of individuals present inside the colony area.

Although there is an increasing body of literature supporting the effectiveness of UAVs as a future monitoring tool in wildlife ecology, it is important to establish best-practice protocols for the different types of tasks, especially in protected areas, and it is highly relevant for more studies on how to improve data analysis to optimize the effort invested in monitoring.

## Figures and Tables

**Table 1:** Results of the different linear models fitted for the best conversion factors on both flights (15 and 30 May). Best models are considered the ones with best fitted Ajd R^2^ and slopes closer to 1.

Date of the Flight	Species	Conversion Factor	Adj R^2^	Slope	S.E.	t	*p*
15 May	HG	0.7	0.74	0.99	0.12	8.36	<0.001
	LBBG	0.7	0.96	0.97	0.04	24.66	<0.001
30 May	HG	0.6	0.88	0.94	0.06	14.70	<0.001
	LBBG	0.7	0.99	0.94	0.01	70.63	<0.001

**Table 2:** Mean number of individuals of HG and LBBG present in two sections of the colony within 2 h periods from 04:20 to 06:00 h. ‘N’ is the number of 20 min intervals where a photo was taken during the 2 (HG) or 3 (LBBG) days of survey.

Time Interval (h)	Herring Gull			Lesser Black-Backed Gull		
	Average	SD	N	Average	SD	N
04:20–06:00	14.17	1.90	12	17.44	1.85	18
06:20–08:00	14.25	1.71	12	17.00	2.30	18
08:20–10:00	12.75	2.26	12	15.83	2.77	18
10:20–12:00	13.20	4.94	10	15.83	6.40	18

**Table 3:** Outputs from the test of changes in numbers of birds present in the two colony plots during the first part of the day. Date was included as a random effect and the model corrected for a first-order autocorrelation. Photos of the two plots were taken by the wildlife cameras at 20 min intervals.

	Herring Gull				Lesser Black-Backed Gull			
Effect	df	F	*p*	Estimate	df	F	*p*	Estimate
timeseq	1.42	3.69	0.0614	−0.4727	1.62	0.39	0.5360	0.1504
timeseq*timeseq	1.42	3.21	0.0806	0.01734	1.62	1.32	0.2538	−0.0108
Intercept	-	-	-	159.935	-	-	-	16.457

**Table 4:** Individuals counted (in total) and estimated number of breeding pairs (factor 0.7) using UAVs and traditional methods (ground counts of individuals) on the island of Langli during the period of study. Traditionally, the highest number of birds counted on the different ground counts is used as a survey result for the year.

Species	Method	Date	Number of Birds	Estimated n. of Breeding Pairs
Herring Gull	UAV (E384 fixed wing)	15 May	17,144	12,001
	UAV (E384 fixed wing; estim.) Ground count 1 Ground count 2	30 May 28 May 2 June	16,414 8655 10,813	11,490 6059 7569
Lesser Black-backed Gull	UAV (E384 fixed wing)	15 May	3114	2180
	UAV (E384 fixed wing; estim.) Ground count 1 Ground count 2	30 May 28 May 2 June	2651 1723 913	1856 1206 639

## Data Availability

The size of the data collected in terms of drone imagery does not allow authors to store it online. However, these data are available from the corresponding authors on request.
